# High fluoride and low pH level have been detected in popular flavoured beverages in Malaysia

**DOI:** 10.12669/pjms.302.4413

**Published:** 2014

**Authors:** Zubaidah HA Rahim, Marina M Bakri, Zakir HM, Ahmed IA, Zulkifli NA

**Affiliations:** 1 Zubaidah HA Rahim, BSc, PhD, Department of Oral Biology & Biomedical Sciences, Faculty of Dentistry, University of Malaya, 50603 Kuala Lumpur, Malaysia.; 2 Marina M Bakri, BDS, PhD, Department of Oral Biology & Biomedical Sciences, Faculty of Dentistry, University of Malaya, 50603 Kuala Lumpur, Malaysia.; 3 Zakir HM, BDS, PhD, Department of Oral Biology & Biomedical Sciences, Faculty of Dentistry, University of Malaya, 50603 Kuala Lumpur, Malaysia.; 4 Ahmed IA, BDS, Department of Oral Biology & Biomedical Sciences, Faculty of Dentistry, University of Malaya, 50603 Kuala Lumpur, Malaysia.; 5 Zulkifli NA, BDS, Department of Oral Biology & Biomedical Sciences, Faculty of Dentistry, University of Malaya, 50603 Kuala Lumpur, Malaysia.

**Keywords:** Beverages, Dental Erosion, Dental Fluorosis, Fluoride, pH

## Abstract

***Objective:*** In children, excessive ingestion of fluoride from different sources including bottled drinking water and flavoured beverages or soft drinks can lead to the development of dental fluorosis. In addition, the pH level of beverages is important. Low pH can cause dental erosion. In this study we explore the fluoride content and pH level of certain popular beverages available in Malaysian supermarkets and hawkers’ stalls.

***Methods:*** Bottled drinking water and selected popular flavoured packet drinks were purchased from a supermarket and the corresponding flavoured hawkers’ drinks, from a hawker’s stall in Kuala Lumpur. Fluoride and pH of the beverages were determined using digital fluoride meter and digital pH meter respectively.

***Results:*** It was found that fluoride content and pH level vary among the beverages. The mean fluoride content in both packet and hawkers’ drinks (7.64±1.88 mg/L, 7.51±1.60 mg/L, respectively) was approximately 7 times higher than the bottled drinking water (1.05±0.35 mg/L). Among the beverages, the tea packet drink was found to contain the highest amount of fluoride (13.02±0.23 mg/L). The mean pH of bottled-drinking water was near neutral (6.96±0.17), but acidic for both supermarket (4.78.00±0.49) and hawkers’ drinks (5.73±0.24). The lychee packet drink had the lowest pH level (2.97±0.03).

***Conclusions:*** Due to the wide variation of the fluoride content and pH level of the drinks tested in this study, it is recommended that steps should be taken to control the fluoride concentration and pH level in beverages if dental fluorosis and erosion are to be prevented.

## INTRODUCTION

Optimal intake of fluoride lowers the decay of teeth.^1^ A decline in dental caries in the developed countries has been attributed to the widespread use of systemic and topical fluorides. The topical sources of fluoride are mouth rinses, tooth pastes while systemic sources can be community drinking water, beverages and foods.^1^^,^^2^ Water fluoridation has been implemented successfully to supply the fluoride to the community.^2^ In Peninsula Malaysia, water fluoridation has been accepted as a government policy in 1972.^3^ The recommended level of water fluoridation by the Ministry of Health, Malaysia, is 0.5 – 0.9 mg/L.^3^ World Health Organization (WHO) recommended the range of 0.7-1.2 mg/L.^4^ However, excess ingestion of fluoride can lead to mottling of enamel or dental fluorosis and fluoride toxicity.^5^^,^^6^ Higher cases of dental fluorosis amongst the population in Kelantan, Terengganu and Sabah in Malaysia have been reported.^5^ Fluorosis in enamel and even in dentine is caused by the ingestion of excess fluoride during tooth development.^5^^,^^6^ In children, exposure to high level of fluoride during tooth development can lead to dental fluorosis.^7^ As fluoride is available in different sources (like drinking water, foods, fruits, beverages) a person in modern life can easily be exposed to fluoride which may be higher than the recommended dose. Changes in lifestyle promote the increased use of beverages among the people of all ages including children all over the world. Increased consumption of fluoridated beverages along with fluoridated water increases the risk of dental fluorosis.

Beverages which are acidic in nature are also detrimental to teeth as acidity can lead to dissolution of the dental structure.^8^^,^^9^ The pH seven is neutral and less than seven is acidic while more than seven is alkaline. Some beverages contain high concentration of citric acid, phosphoric acid and other dietary acids which contribute to lower pH.^9^^,^^10^ It has been found that the frequent use of beverages is associated with increasing dental erosion.^11^

Therefore, exploration of the fluoride content and pH level in popular beverages is important in preparing the safe level guidelines of fluoride concentration and pH level in beverages. In Malaysia, fluoride content and pH level in various types of beverages especially amongst the popular beverages have not been reported. In the present study our objective was to explore the fluoride content and pH level in certain popular beverages available in Malaysian supermarket and hawkers’ stalls.

## METHODS

In this study popular beverages chosen were categorized as a) bottled-drinking water, b) packet drinks and c) hawkers’ drinks. Bottled and packet drinks were purchased from a supermarket and hawkers’ drinks were purchased from one hawker’s stalls in Kuala Lumpur. Four popular flavours were chosen from packet drinks and hawkers’ drinks which were soya bean, lychee, sugarcane, and green tea. Four different brands of bottled-drinking water were also used. Three different samples of each type of beverages were used in the study. For each sample the fluoride content and pH level were measured in triplicate.

***Fluoride Content Determination:*** A photometer (HANNA instrument; model no: HI 93739; USA) was used for measuring fluoride. The principles of this fluoride meter are based on adaptation of SPADNS (sodium 2-(parasulfophenylazo)-1,8-dihydroxy-3,6-naphthalenedisulfonate) method. Light source for this model is light emitting diode and the light detector is silicon photocell. This method is approved by the U.S. Environmental Protection Agency. Fluoride ion specific electrode (model no. 9609BN, Orion, USA) was used to detect fluoride content for the coloured beverages. A calibration was done using known standards of fluoride before measuring the fluoride content of beverages. Procedure was carried out according to the company standard manual. These instruments were used to detect fluoride by previous researchers.^12^^,^^13^

***pH determination:*** Ten milliliter of samples from beverages was taken into a beaker and pH level of samples was measured using a Denver Instrument Benchtop (model no:UB-10; USA). Standard buffers, pH4.0 and pH 7.0 were used for calibration. The pH measurements were carried out in a common place and common room temperature (27ºC) to minimize the environmental effect on pH. To minimize measurement errors all instruments in this experiment were washed using distilled and deionized water and dried with clean tissue paper after each reading.

***Data Analysis:*** Mean and standard deviations of fluoride and pH level for all samples were calculated. One way ANOVA followed by Tukey’s test was performed to see the difference among the drinking water, packet drinks and hawkers’ drinks. A *p*-value <0.05 was taken as statistically significant.

## RESULTS

***Fluoride Level:*** The fluoride concentration appeared to vary among the beverages tested (Table-I). The range of fluoride in bottled drinking water was 0.211± 0.08 mg/L to 2.633± 0.17 mg/L. All packet drinks except soy bean were found to have high fluoride concentration. Highest concentration of fluoride was found in tea-packet drink (13.02 ± 0.23 mg/L). Hawkers’ drinks also have high concentration of fluoride except for soy bean. Among the hawkers’ drinks, sugarcane was found to contain highest concentration (10.922 ± 0.92 mg/L).

**Fig.1:**
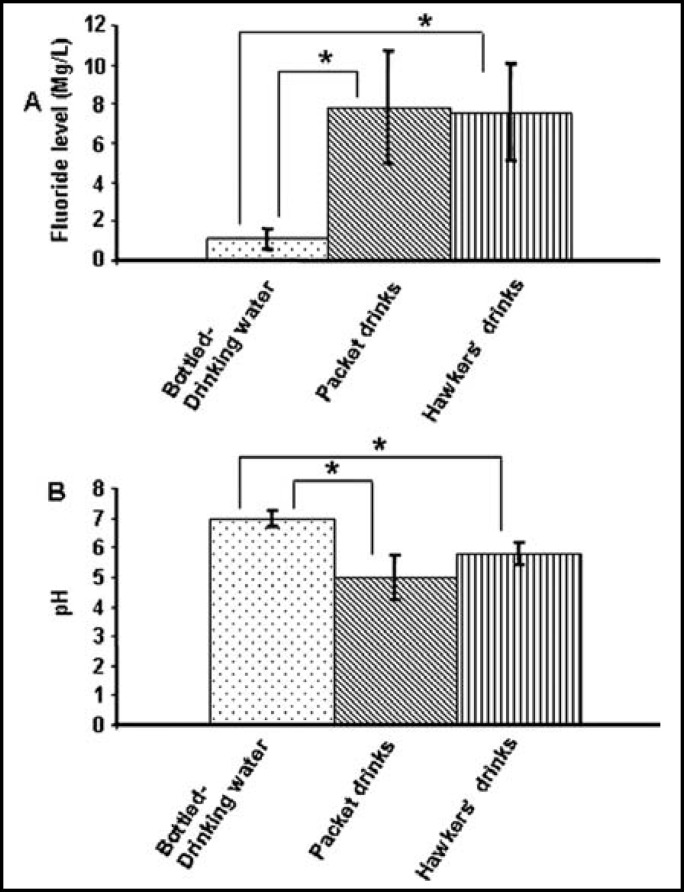
Comparison of fluoride content (A) and pH level (B) among the different categories of beverages

Upon comparison among the bottled-drinking water, packet and hawkers’ drinks it was found that the packet and hawkers’ drinks contain around 7 times higher fluoride level than bottled-drinking water (Fig.1A).

***pH Level:*** The pH level was also found to vary among the beverages tested (Table-I). The pH level in bottled-drinking water was around neutral level (6.22 ± 0.04 to 7.46 ± 0.02). Some packet and hawkers’ drinks were in the acidic range. Among the packet drinks, the lychee was found to have the lowest pH (2.97 ± 0.03). Among the hawkers’ drinks, sugarcane was found to have low pH (5.11 ± 0.02).

Comparing the pH level among the different categories of beverages, it was found that both the packet and hawkers’ drinks have significantly lower pH than the bottled-drinking water (Fig.1B). Packet drinks have lower pH level than hawkers’ drinks although the difference was not significant.

## DISCUSSION

The present study for the first time explores fluoride content and pH level in popular beverages available in Malaysian supermarket and hawkers stall. Our study reveals high fluoride content and low pH level in certain popular beverages in Malaysia.

**Table-I:** Fluoride content and pH level in popular beverages in Malaysia

*Beverages*	*Fluoride (mg/L)* *(mean ± SD)*	*pH (mean ± SD)*
*1. Bottled-drinking water*		
Bottled-drinking water-1	0.52 ± 0.11	6.85 ± 0.04
Bottled-drinking water-2	1.08 ± 0.34	7.36 ± 0.03
Bottled-drinking water-3	2.63 ± 0.1658	7.46 ± 0.02
Bottled-drinking water-4	0.21 ± 0.0781	6.22 ± 0.04
*2. Packet drinks*		
Soy bean	0.41 ± 0.02	6.55 ± 0.02
Lychee	11.64 ± 0.16	2.97 ± 0.03
Sugarcane	6.20 ± 0.13	4.89 ± 0.02
Tea	13.02 ± 0.23	5.60 ± 0.03
*3. Hawkers’ drinks*		
Soy bean	0.537 ± 0.01	6.63 ± 0.02
Lychee	10.82 ± 0.28	6.29 ± 0.23
Sugarcane	10.92 ± 0.92	5.11 ± 0.02
Tea	8.02 ± 0.23	5.18 ± 0.05

Note that fluoride content in packet and hawkers’ drinks was around 7 times higher than bottled-drinking water. * p<o.o5, (One-way ANOVA followed by Tukey’s test).

Our result of high concentration of fluoride in beverages is in accordance with previous studies conducted in other countries.^13^^-^^16^ In a study conducted in ready to drink fruit- juices in the USA by Stannard et al. 1991,^14^ showed that the fluoride level in juices were 0.15 to 6.80 mg/L and 42% of their studied fruit juices contain more than 1 parts per million (1ppm =1 mg/L) of fluoride. A study carried out by a group of researcher in New Zealand showed that the fluoride level in juices and juice-flavored drinks ranged from 0.02 to 2.80 parts per million.^15^

In our study we observed that fluoride level in tea and lychee drinks was very high both for packet and hawkers’ drinks. High fluoride level in tea was also found in other countries.^13^^,^^16^ Fluoride content ranged from 0.12 to 8.33 ppm in commercial tea has been reported in a study in Poland.^13^ In a study conducted in Turkey, fluoride content in black tea was found from 0.57 to 3.72 mg/L.^16^ Results of the studies including ours indicate that fluoride content varies with the type of beverages. The reason may be the source of water that is used for making the beverages. Fluoride level in water shows geographical variation.^3^^,^^12^ The study shows different level of fluoride concentration in water in different regions in Malaysia^3^ The difference may also be due to the difference in fluoride concentration in various fruits.^14^ Future study to find the normal level of fluoride in the original produce – i.e. soy bean, raw sugarcane stems, lychee fruit and tea can be beneficial to understand the finding of our study. Ingestion of excessive fluoride during infancy and early childhood may cause dental fluorosis of permanent anterior teeth which are the most aesthetically important teeth.

High fluoride exposure also has an adverse effect on children’s neurodevelopment.^17^ Fluoride was found to cause neurotoxicity in laboratory animals, including effects on learning and memory.^18^ Fluoride can cross the placenta. Therefore, a mother taking high fluoride may affect developing teeth during the intrauterine life. The presence of high fluoride concentration highlights the importance of continuous monitoring of fluoride levels in beverages. Listing fluoride content of beverages would be desirable. For clinicians, the knowledge about possible fluoride ingestion from dietary sources is important when recommending the safest schedule of fluoride treatment to reduce the risk of dental fluorosis. Indeed, the U.S. Department of Health and Human Services has proposed to change the recommended level of fluoride in drinking water to 0.7 mg/L from the currently recommended range of 0.7–1.2 mg/L, and the Environmental Protection Agency is reviewing the maximum amount of fluoride allowed in drinking water, which currently is set at 4.0mg/L.^19^

In our study the pH in packet and hawkers’ drinks was found to be significantly lower compared to bottled-drinking-water. In Malaysia, hawkers’drinks and bottled drinks respectively are popular among the patrons of stalls and restaurants during lunch or dinner. Packet drinks were relatively more acidic than hawkers’ drinks although the difference was not statistically significant. The most likely reason for this observation is the dilution of drinks by hawkers. During the manufacturing of packet drinks citric acid, phosphoric acid and other types of acid is added that may cause their high acidity.^9^^,^^20^ We found very low level of pH in lychee packet drinks. The low pH in consumed beverages has been reported in a study carried out in Iran.^21^ pH in around neutral level (6.2-7) has no destructive effect on teeth. Critical pH when dental erosion starts to occur is around 5.5.^21^^,^^22^ A study showed that the degree of enamel erosion initiated by marketed fruit juice was about 5-8 times higher than that of the minced fruit juice.^23^

Many studies on children and adolescents have concluded that acidic drinks cause tooth erosion.^11^^,^^24^^-^^26^ A study conducted in Saudi Arabia showed the presence of significant correlation between the number of maxillary incisors with pronounced erosion on their palatal surfaces and the frequency of taking beverage drinks at night.^11^ This correlation between the frequency of taking beverages and erosion of teeth was found in both primary and permanent teeth.^11^ It has been reported that the frequent and excessive intake of beverages with low pH is detrimental to teeth.

## CONCLUSION

The high concentration of fluoride and low level of pH in popular beverages in Malaysia found in this study suggest the necessity of monitoring of fluoride and pH level in beverages by the authority in order to prevent dental fluorosis and erosion especially in children.

## Authors Contribution:

**ZHR:** Designed the protocol, interpreted the data, reviewed & approved the manuscript.

**MMB & ZHM:** Analyzed and interpreted the data, wrote the manuscript, revised the manuscript critically. 

**AIA & ZNA:** Conceived the idea, did data collection & analysis.

## Notes

***Source of Funding: ***PPP grant University of Malaya.
